# Daikenchuto, a Japanese herbal medicine, ameliorates experimental colitis in a murine model by inducing secretory leukocyte protease inhibitor and modulating the gut microbiota

**DOI:** 10.3389/fimmu.2024.1457562

**Published:** 2024-10-25

**Authors:** Sotaro Ozaka, Akira Sonoda, Yoko Kudo, Kanako Ito, Naganori Kamiyama, Nozomi Sachi, Thanyakorn Chalalai, Yomei Kagoshima, Yasuhiro Soga, Supanuch Ekronarongchai, Shimpei Ariki, Kazuhiro Mizukami, Shiori Ishizawa, Mitsue Nishiyama, Kazunari Murakami, Kiyoshi Takeda, Takashi Kobayashi

**Affiliations:** ^1^ Department of Infectious Disease Control, Faculty of Medicine, Oita University, Yufu, Japan; ^2^ Department of Gastroenterology, Faculty of Medicine, Oita University, Yufu, Japan; ^3^ Tsumura Advanced Technology Research Laboratories, Research and Development Division, Tsumura & Co., Inashiki, Japan; ^4^ Laboratory of Immune Regulation, Department of Microbiology and Immunology, Graduate School of Medicine, WPI Immunology Frontier Research Center, Osaka University, Suita, Japan; ^5^ Research Center for GLOBAL and LOCAL Infectious Diseases, Oita University, Yufu, Japan

**Keywords:** Daikenchuto (DKT), SLPI, DSS-induced colitis, inflammatory bowel disease (IBD), butyric acid, *Parabacteroides*, *Allobaculum*, *Akkermansia*

## Abstract

**Background:**

Inflammatory bowel disease (IBD) is a refractory inflammatory disorder of the intestine, which is probably triggered by dysfunction of the intestinal epithelial barrier. Secretory leukocyte protease inhibitor (SLPI) secreted by colon epithelial cells protects against intestinal inflammation by exerting anti-protease and anti-microbial activities. Daikenchuto (DKT) is one of the most commonly prescribed Japanese traditional herbal medicines for various digestive diseases. Although several animal studies have revealed that DKT exerts anti-inflammatory effects, its detailed molecular mechanism is unclear. This study aimed to clarify the anti-inflammatory mechanism of DKT using a murine colitis model, and to evaluate its potential as a therapeutic agent for IBD.

**Methods:**

Experimental colitis was induced in wild-type (WT) mice and SLPI-deficient (KO) mice by dextran sulfate sodium (DSS) after oral administration of DKT. The resultant clinical symptoms, histological changes, and pro-inflammatory cytokine levels in the colon were assessed. Expression of SLPI in the colon was detected by Western blotting and immunohistochemistry. Composition of the gut microbiota was analyzed by 16S rRNA metagenome sequencing and intestinal metabolites were measured by gas chromatography-mass spectrometry analysis. Intestinal epithelial barrier function was assessed by oral administration of FITC-dextran and immunostaining of tight junction proteins (TJPs).

**Results:**

Oral administration of DKT increased the number of butyrate-producing bacteria, such as *Parabacteroides*, *Allobaculum*, and *Akkermansia*, enhanced the levels of short-chain fatty acids, including butyrate, in the colon, induced SLPI expression, and ameliorated DSS-induced colitis in WT mice. We found that mouse colon carcinoma cell line treatment with either DKT or butyrate significantly enhanced the expression of SLPI. Moreover, supplementation of DKT protected the intestinal epithelial barrier with augmented expression of TJPs in WT mice, but not in KO mice. Finally, the composition of the gut microbiota was changed by DKT in WT mice, but not in KO mice, suggesting that DKT alters the colonic bacterial community in an SLPI-dependent manner.

**Conclusion:**

These results indicate that DKT exerts anti-inflammatory effects on the intestinal epithelial barrier by SLPI induction, due, at least in part, to increased butyrate-producing bacteria and enhanced butyrate levels in the colon. These results provide insight into the mechanism of the therapeutic effects of DKT on IBD.

## Introduction

Inflammatory bowel disease (IBD), including ulcerative colitis (UC) and Crohn’s disease (CD), is a recurrent inflammatory disorder of the gastrointestinal tract that occurs in young people. It is recognized as a global health problem because the incidence and prevalence of IBD are rapidly increasing worldwide (1, 2). However, the pathogenesis of IBD remains largely unknown, and no fundamental treatment has been developed for it. In recent years, molecular targeted drugs targeting various cytokines, integrins, and Janus kinase (JAK) have been developed to treat patients with refractory IBD with an inadequate response to existing therapies, such as 5-aminosalicylic acids, immunomodulators, and corticosteroids (3). While these drugs are effective in treating refractory IBD, their immunosuppressive side effects and the high cost of these agents are problematic. Therefore, new therapeutic drugs for IBD with high safety and cost-effectiveness are desired (4).

Daikenchuto (DKT), a Japanese traditional herbal medicine (Kampo medicine), is one of the most commonly prescribed herbal medicines for patients with gastrointestinal disorders, such as constipation and intestinal obstruction. While Kampo medicine was introduced in ancient China and developed uniquely as a traditional medicine in Japan, it is also used in Western countries due to its effectiveness and high safety profile (5, 6). Although the mode of action of DKT was unknown for a long time, a comprehensive analysis of its pharmacological properties has been conducted at component and molecular levels since the 2000s. DKT consists of an aqueous extract powder prepared from a mixture of *Zingiberis rhizoma* (Ginger), *Zanthoxyli fructus* (Japanese pepper), *Panax ginseng* (Ginseng radix), and maltose. DKT has the effects of increasing intestinal blood flow, improving gastrointestinal motility, and inhibiting fibrosis (7–9). In fact, several clinical trials have demonstrated the preventive effect of DKT on intestinal obstruction after abdominal surgery (10, 11). In addition, several animal studies have demonstrated that DKT exerts anti-inflammatory effects (12–14). Moreover, dietary administration of DKT is known to alter the composition of the gut microbiota in mice, suggesting that DKT acts on the host’s immune system by affecting the gut microbiota (15, 16). Shi et al. reported that DKT ameliorates acute experimental colitis by altering gut microbial composition and increasing propionate acids (17). However, the precise mechanisms of its anti-inflammatory effects and the regulatory function of the intestinal microbiota remain largely unclear.

Secretory leukocyte protease inhibitor (SLPI) is an endogenous serine protease inhibitor secreted by glandular tissue that antagonizes neutrophil proteases, such as neutrophil elastase (NE) and cathepsin G (18). Previous studies using SLPI-deficient mice (SLPI^-/-^) have shown the tissue protective effects of SLPI through anti-protease activity in the skin, lung, and colon (19–21). SLPI has also been reported to have antimicrobial activity (22), suggesting that it regulates the gut microbiota by acting as an antimicrobial peptide in the intestine.

The purpose of this study was to elucidate the anti-inflammatory mechanism of DKT and potential involvement of SLPI using a murine colitis model. Here, we showed that oral administration of DKT induced the expression of SLPI, in association with an increase in the number of butyric acid-producing bacteria in the colon of mice, and administration of DKT ameliorated the disease severity of DSS-induced colitis in an SLPI-dependent manner. These results provide insight into the possible mechanisms of the therapeutic effects of DKT on IBD.

## Results

### DKT ameliorates DSS-induced colon inflammation

**Figure 1:**
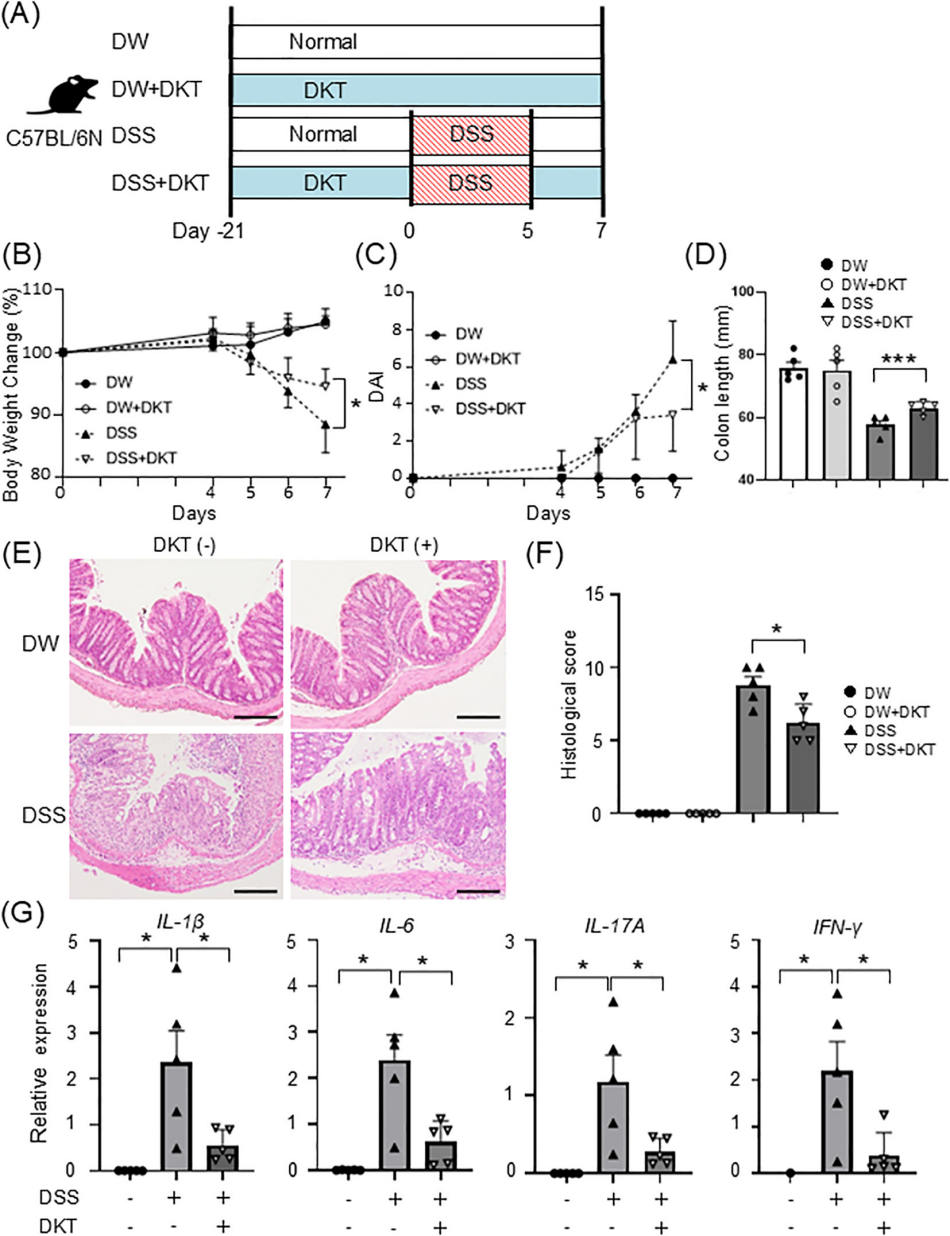
DKT ameliorates DSS-induced colon inflammation in wild-type (WT) mice. **(A)** Experimental schematic: a normal water and diet group (DW), normal water and DKT diet group (DW+DKT), DSS-induced colitis group (DSS), and DSS-induced colitis with DKT treatment group (DSS+DKT) were created using C57BL/6N mice. **(B)** Changes in body weight in WT mice were monitored for seven days after DSS treatment (n = 5 in each group). **(C)** Clinical symptoms of DSS-induced colitis were determined by the disease activity index (DAI) (n = 5 in each group). **(D)** Colon length was measured on day 7 (n = 5 in each group). **(E)** The distal colon was excised on day 7, sectioned, and stained with H&E. Scale bars represent 100 μm. **(F)** Histological scores were evaluated on day 7 (n = 5 in each group) **(G)** mRNA expression levels of the indicated pro-inflammatory cytokines were determined by quantitative RT-PCR (n = 5 in each group). Statistical analysis was performed using one-way ANOVA followed by Tukey’s multiple comparisons test. *: *P* < 0.05 and ***: *P* < 0.001.

To examine the efficacy of DKT on intestinal inflammation, we first compared the disease severity of DSS-induced colitis with and without DKT in wild-type (WT) mice. We prepared the following four experimental groups: a normal water and diet group (DW), normal water and DKT diet group (DW+DKT), a DSS-induced colitis group (DSS), and a DSS-induced colitis with DKT treatment group (DSS+DKT) (**Figure 1A**). Based on the study group, mice were given a normal diet or a 3.6% DKT diet for 21 days, followed by 2% DSS for 5 days, as appropriate. Although the body weight of the DW and DW+DKT groups increased slightly, both DSS and DSS+DKT groups demonstrated weight loss, diarrhea, and bloody feces. As shown in **Figures 1B, C**, however, the weight loss and colitis symptoms in DSS+DKT mice were significantly suppressed on day 7 compared to the DSS group. It is generally accepted that a decrease in colon length in DSS-treated mice is associated with the severity of inflammation and fibrosis in experimental colitis (23). Colon length in the DSS+DKT group was significantly longer than that in the DSS group (**Figure 1D**). Histological analysis showed that severe crypt loss and inflammatory cell infiltration in the colonic mucosa were seen in the DSS group, while these symptoms were significantly milder in the DSS+DKT group (**Figures 1E, F**). Furthermore, mRNA levels of IL-1β, IL-6, IL-17A, and IFN-*γ* were significantly up-regulated in the colon of the DSS group compared to the control DW group. The expression levels of these pro-inflammatory cytokines in the colon were significantly suppressed in the DSS+DKT group compared to the DSS group (**Figure 1G**). These results indicate that DKT treatment ameliorates DSS-induced colon inflammation in WT mice.

### DKT induces a high expression of SLPI in intestinal epithelial cells and modulates the gut microbiota or metabolites

**Figure 2:**
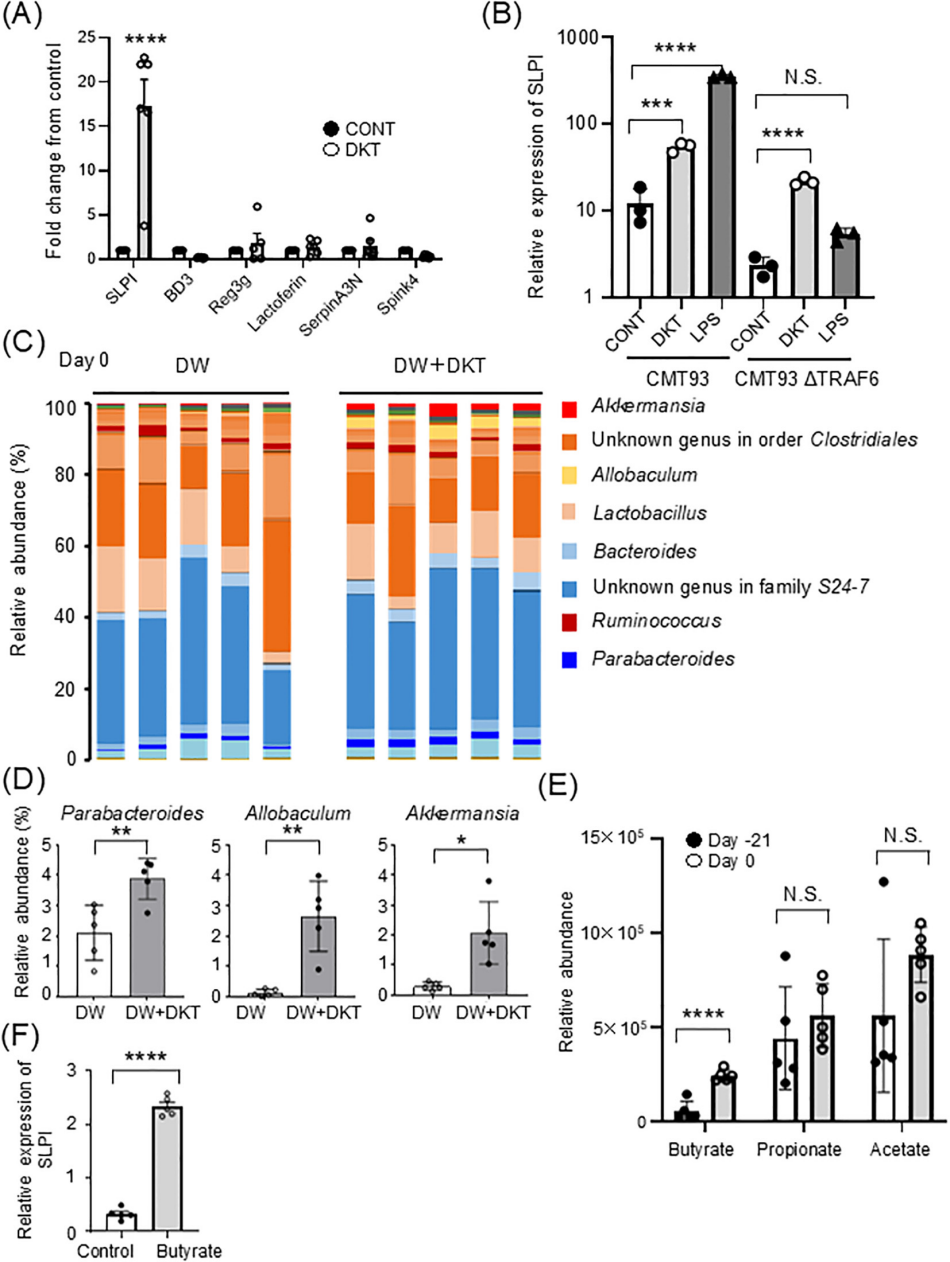
DKT alters the gut environment and induces SLPI in intestinal epithelial cells. **(A)** Quantitative RT-PCR analysis of the mRNA expression of *SLPI* and other antimicrobial peptides or protease inhibitors in DKT extract powder (DKT-E)-treated CMT93 cells. Cells were treated with DKT-E (1 mg/mL) for 24 hours. Relative mRNA expression of the indicated genes in CMT93 cells treated with DKT-E is shown as the fold-increase compared to untreated control cells (CONT). Graphs show the mean ± SEM (n = 6). **(B)** Quantitative RT-PCR analysis of the mRNA expression of *SLPI* in CMT93 and TRAF6-deficient CMT93 cells (CMT93ΔTRAF6). Cells were treated with DKT-E (1 mg/mL) or LPS (3 μg/mL) for 24 hours, or were left untreated (CONT). Graphs show the mean ± SEM (n = 3). **(C)** Fecal samples from mice fed a normal diet (DW) or DKT diet (DW+DKT) for 21 days (on day 0) were subjected to 16S rRNA metagenome sequencing to examine the composition of the gut microbiota. The relative abundance of bacterial genera is shown. Each bar shows relative bacterial abundance in individual mice (n = 5 in each group). **(D)** Relative bacterial abundance is shown at the genus level. Data are presented as the mean ± SEM (WT: n = 5, WT+DKT: n = 5) **(E)** The relative concentrations of the indicated SCFAs in murine feces before (Day -21) and after (Day 0) DKT administration. Stool samples from the same mice were analyzed by GC/MS. **(F)** CMT93 cells were stimulated with sodium butyrate (1 mM) for 24 hours. Relative mRNA expression of *SLPI* is shown. Statistical analysis was performed using one-way ANOVA followed by Tukey’s multiple comparisons test or student’s *t* test. Data are expressed as the mean ± SEM (n = 5 in each group). *: *P* < 0.05, **: *P* < 0.01, ***: P < 0.001, ****: *P* < 0.0001, and NS, not significant.

To clarify the protective mechanisms of DKT in the intestine, we examined whether DKT induces colon protective molecules, such as antimicrobial peptides or protease inhibitors. CMT93 cells, a colon carcinoma cell line, were cultured for 24 hours in the presence or absence of DKT extract powder (DKT-E), and mRNA expression was examined. As shown in **Figure 2A**, mRNA expression of *SLPI* was significantly increased in CMT93 cells treated with DKT-E compared to that in untreated control cells, which was more pronounced than other protease inhibitors, including β-defensin, regenerating islet-derived protein IIIγ (Reg-IIIγ), and lactoferrin or protease inhibitors, such as serine protease inhibitor A3N (SerpinA3N) and serine peptidase inhibitor kazal type 4 (Spink4). We previously reported that lipopolysaccharide (LPS) stimulation induces *SLPI* expression (21) (**Figure 2B**, left), which depends on the TLR signaling pathway through TRAF6, since CMT93 cells lacking TRAF6 failed to induce *SLPI* (**Figure 2B** right). Interestingly, however, induction of SLPI expression by DKT-E treatment, albeit weaker than that induced by LPS (**Figure 2B**, left), occurred even in the absence of TRAF6 (**Figure 2B**, right), suggesting that DKT-E induces SLPI from intestinal epithelial cells in a TLR ligand-independent manner. Since the gut microbiota is an important factor in the severity of colitis (24), we performed 16S rRNA metagenome sequencing analysis of stool samples of mice treated with DKT. In the gut microbiota composition profile, the abundance of beneficial bacteria, such as *Parabacteroides*, *Allobaculum*, and *Akkermansia*, were increased at the genus level in WT mice after treatment with DKT (day 0) (**Figures 2C, D**). Since these species of bacteria are known to produce short chain fatty acids (SCFAs), we next analyzed SCFAs in fecal samples using GC/MS. As expected, dietary DKT increased SCFAs, including butyrate, propionate and acetate in fecal samples (**Figure 2E**). Notably, butyrate, a major gut microbiome-derived SCFA, was significantly higher in WT mice treated with DKT. Recent studies have demonstrated that butyrate might exert beneficial effects on barrier integrity by inducing endogenous antimicrobial peptides (25). Interestingly, the expression of *SLPI* mRNA in CMT93 cells was significantly increased after 24 hours of butyrate treatment compared to that in untreated control cells *in vitro* (**Figure 2F**). Similarly, oral supplementation of butyrate slightly, but not significantly, increased SLPI expression in the mice colon (**Supplementary Figure 1A**). These results suggest that DKT enhances SLPI expression directly in the colon, as well as indirectly by modulating the gut microbiota and increasing butyrate production.

### DKT induces SLPI in the intestine *in vivo* and attenuates DSS-induced colitis in WT mice, but not in SLPI^-/-^ mice

**Figure 3:**
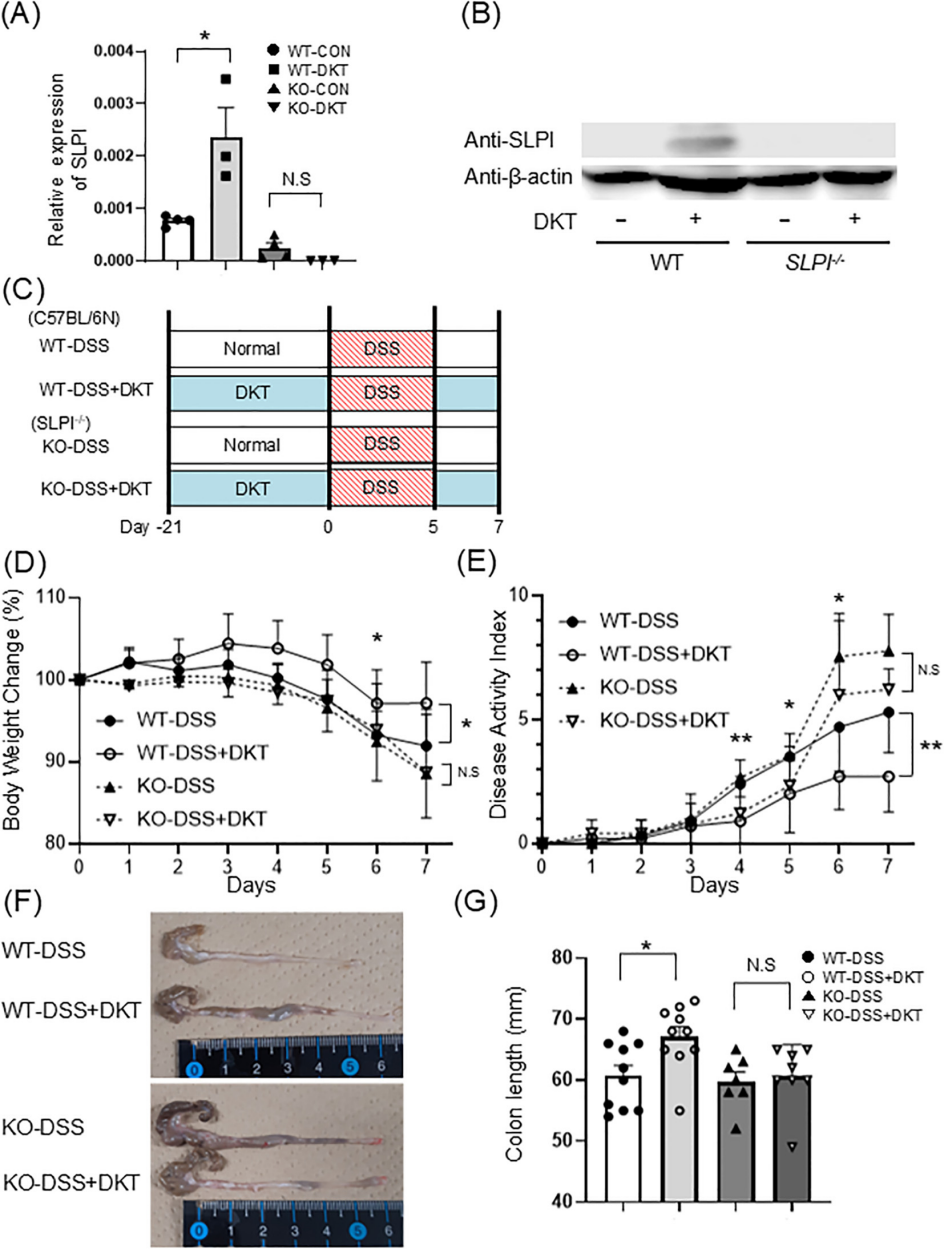
Induction of SLPI is required for the suppressive effect of DKT on DSS-induced colitis. **(A)** Quantitative RT-PCR analysis of the mRNA expression of *SLPI* in the colon of mice after the treatment of DKT. Graphs show the mean ± SEM (n = 3-4). **(B)** SLPI protein in colonic tissues of WT and KO mice with (+) and without (-) DKT. SLPI was detected by Western blotting. **(C)** Experimental schematic: WT and SLPI^-/-^ (KO) mice were administered DSS after pre-feeding with a normal diet (DSS) or a DKT diet (DSS+DKT). **(D)** Body weight changes in WT and SLPI^-/-^ mice were monitored after DSS treatment (WT-DSS group: n = 10, WT-DSS+DKT group: n = 10, KO-DSS group: n = 7, KO-DSS+DKT group: n = 8). **(E)** Clinical symptoms of DSS-induced colitis in WT (n = 10) and SLPI^-/-^ mice (n = 9) were determined by the DAI **(F, G)**. Colon length was compared between DSS-treated WT and SLPI^-/-^ mice on day 7 (WT-DSS group: n = 10, WT-DSS+DKT group: n = 10, KO-DSS group: n = 7, KO-DSS+DKT group: n = 8). Data from two different experiments are presented together. Statistical analysis was performed using one-way ANOVA followed by Tukey’s multiple comparisons test. Data represent the mean ± SEM. *: *P* < 0.05 and **: *P* < 0.01. N.S, not significant.

Next, we prepared the following four experimental groups to check the expression of SLPI *in vivo*: WT mice on a normal diet (WT-CON), WT mice on a DKT diet (WT-DKT), SLPI^-/-^ mice on a normal diet (KO-CON), and SLPI^-/-^ mice on a DKT diet (KO-DKT). After 28 days of feeding, *SLPI* mRNA levels were significantly enhanced in the colon of WT-DKT mice, but not in KO-DKT mice (**Figure 3A**). In agreement with this finding, SLPI protein was clearly detected in the colon of DKT-treated WT mice by Western blotting (**Figure 3B**). Furthermore, we investigated the localization of SLPI by immunohistochemistry. SLPI expression was not observed in colon of mice given normal diet (WT-CON), while SLPI expression was observed in goblet cells of the colon of mice treated with DKT (WT-DKT) (**Supplementary Figure 1B**). These results indicate that DKT induces SLPI production.

To clarify the role of SLPI in the suppressive effect of DKT on DSS-induced colitis, we next evaluated the effect of DKT on DSS-induced colitis in WT and SLPI^-/-^ mice. Diet-incorporated DKT was administered from 21 days prior to DSS treatment to the end of the experiment. We prepared the following four experimental groups: WT mice on a normal diet (WT-DSS), WT mice on a DKT diet (WT-DSS+DKT), SLPI^-/-^ mice on a normal diet (KO-DSS), and SLPI^-/-^ mice on a DKT diet (KO-DSS+DKT) (**Figure 3C**). DSS-treated mice continued to experience a decrease in body weight from day 5 of DSS administration to the end of the experiment. Oral DKT administration significantly suppressed weight loss in WT mice, although it failed to do so in SLPI^-/-^ mice (**Figure 3D**). The same was true for the DAI. DKT treatment reduced DAI scores in WT mice, but not in SLPI^-/-^ mice (**Figure 3E**). Moreover, DSS-treated mice displayed a reduction in colon length and grossly thickened walls, which was clearly suppressed by DKT in WT mice, but not in SLPI^-/-^ mice (**Figures 3F, G**). These results suggest that DKT attenuates the severity of DSS-induced colitis in an SLPI-dependent manner.

### DKT treatment ameliorates DSS-induced intestinal inflammation in WT mice, but not in SLPI^-/-^ mice

**Figure 4:**
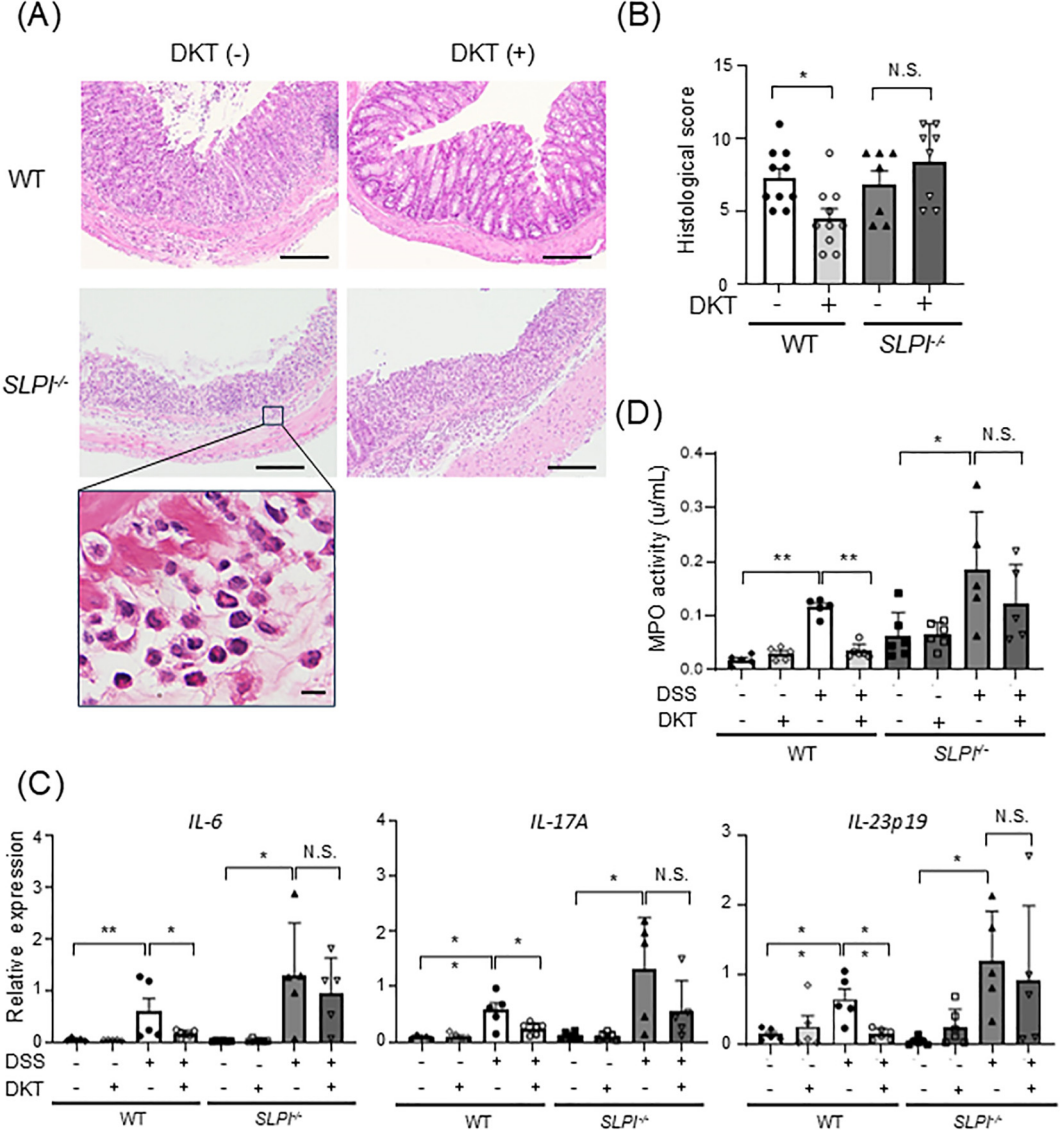
DKT treatment ameliorates DSS-induced intestinal inflammation in WT mice, but not in SLPI^-/-^ mice. **(A)** H&E staining of DSS-induced colitis in WT and SLPI^-/-^ mice treated with DKT (DKT +) or left untreated (DKT -). A higher magnification image of the rectangle in the left panel is shown in the lowest panel. The scale bar represents 100 μm (upper panels) and 10 μm (lowest panel), respectively. **(B)** Graph showing the histological score (WT-DSS group: n = 10, WT-DSS+DKT group: n = 10, KO-DSS group: n = 7, KO-DSS+DKT group: n = 8). **(C)** The mRNA expression level of pro-inflammatory cytokines was determined by quantitative RT-PCR (WT: n = 5 in each group; SLPI^-/-^: n = 6-8 in each group). **(D)** Neutrophil activity was measured by MPO assay (WT-DW: n = 5, WT-DW+DKT: n = 5, WT-DSS: n = 5, WT-DSS+DKT: n = 6, KO-DW: n = 6, KO-DW+DKT: n = 6, KO-DSS: n = 5, and KO-DSS+DKT: n = 5). Statistical analysis was performed using one-way ANOVA followed by Tukey’s multiple comparisons test. Results are expressed as the mean ± SEM. *: *P* < 0.05, **: *P* < 0.01, and NS, not significant.

Next, we examined histopathological changes in DSS-induced colitis and the efficacy of DKT and found that loss of crypts, submucosal infiltration of inflammatory cells, and ulceration were observed in all groups except the WT-DSS+DKT group (**Figure 4A**). Numerous neutrophils with a segmented nucleus with 2-5 lobes were seen to infiltrate the submucosa (**Figure 4A**, lowest panel). In addition, the histological score was significantly lower in the WT-DSS+DKT group than the WT-DSS group (**Figure 4B**). On the other hand, there were no significant differences between the KO-DSS and KO-DSS+DKT groups. We next assessed the mRNA expression levels of pro-inflammatory cytokines in colonic tissue. mRNA levels of IL-6, IL-17A, and IL-23p19 were significantly up-regulated in the colon of DSS-treated WT and SLPI^-/-^ mice as compared to untreated mice. The expression levels of pro-inflammatory cytokines in the colon of DSS-treated WT mice were significantly suppressed by DKT treatment, while no suppression was observed in SLPI^-/-^ mice (**Figure 4C**). Consistent with neutrophil infiltration, MPO activity was increased by DSS treatment, which was significantly suppressed by the administration of DKT in WT mice (**Figure 4D**). These data indicate that treatment with DKT ameliorates the inflammatory changes induced by DSS in WT mice in an SLPI-dependent manner.

### DKT protects the intestinal epithelial barrier by suppression of neutrophil protease in WT mice, but not SLPI^-/-^ mice

**Figure 5:**
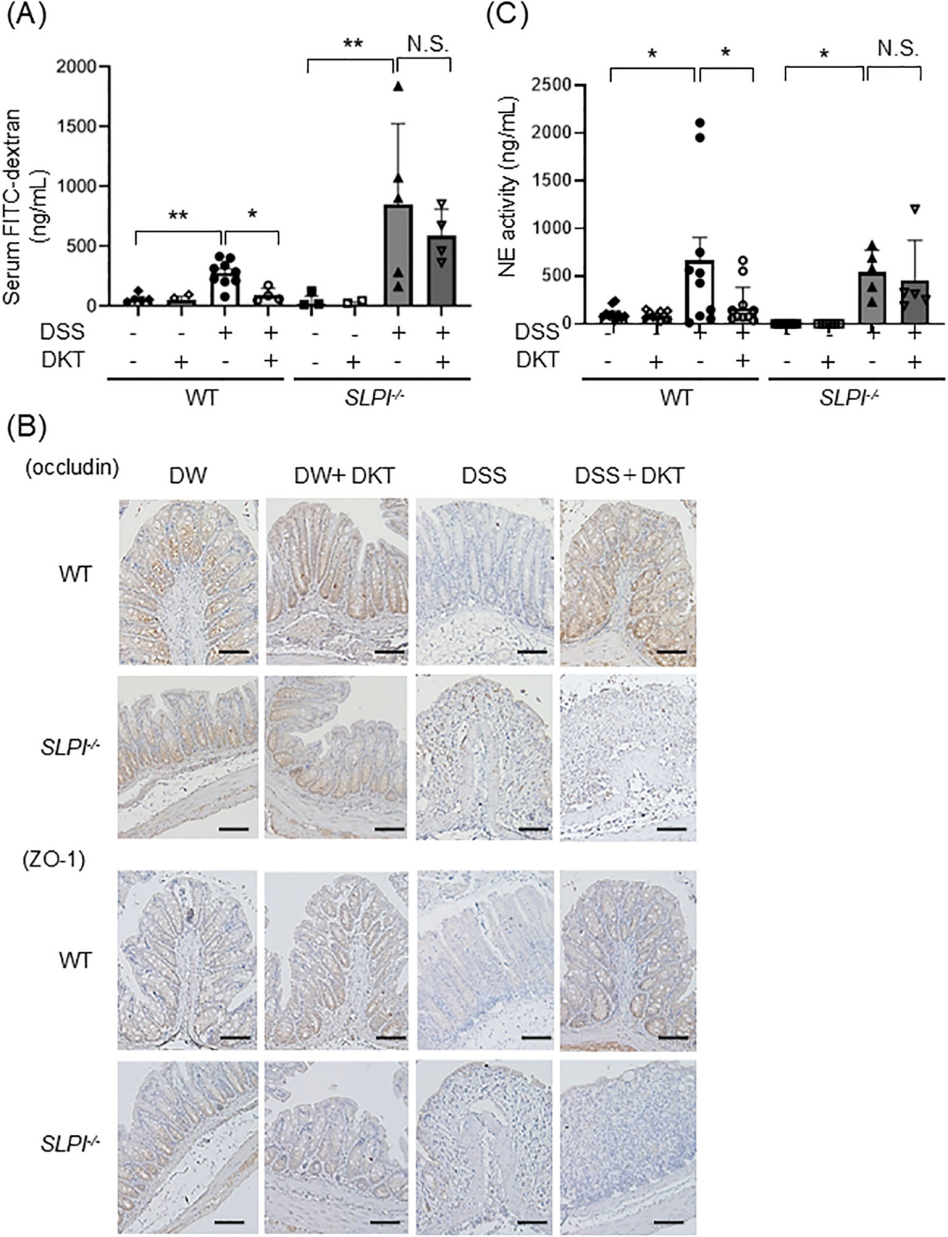
DKT protects the intestinal epithelial barrier by suppression of neutrophil protease in WT mice, but not SLPI^-/-^ mice. **(A)** WT and SLPI^-/-^ mice under the indicated treatment were given FITC-dextran orally, and blood samples were collected 4 hours later. The graph shows FITC-dextran levels in serum in the different groups, determined by fluorescence intensity. Data from two different experiments are presented together. (WT-DW: n = 5, WT-DW+DKT: n = 3, WT-DSS: n = 10, WT-DSS+DKT: n = 4, KO-DW: n = 5, KO-DW+DKT: n = 5, KO-DSS: n = 5, and KO-DSS+DKT: n = 4). **(B)** Expressions of occludin and ZO-1 in the colon tissues from mice under the indicated treatment were detected by immunohistochemistry (×200). Scale bars:100 μm **(C)** Colonic NE activity was measured by NE assay (WT-DW: n = 10, WT-DW+DKT: n = 10, WT-DSS: n = 10, WT-DSS+DKT: n = 12, KO-DW: n = 6, KO-DW+DKT: n = 6, KO-DSS: n = 5, and KO-DSS+DKT: n = 5). Data from two different experiments are presented together. Data were analyzed by one-way ANOVA followed by Tukey’s multiple comparisons test, and are presented as the mean ± SEM. *: *P* < 0.05, **: *P* < 0.01, and NS, not significant.

The intestinal epithelial barrier is essential for intestinal homeostasis, and its dysfunction is implicated in the development of gut inflammation (26). We next examined intestinal epithelial permeability to assess the effects of DKT on the intestinal epithelial barrier in the context of DSS-induced colitis. Mice were given FITC-dextran orally, and intestinal permeability was evaluated by assessing serum FITC-dextran fluorescence levels. Serum FITC-dextran fluorescence levels were comparable at baseline in both DSS and DSS+DKT groups on day 3 because no inflammation was observed in either group (data not shown), indicating that the effect of DKT on the intestinal epithelial barrier function is limited in the early stage of colonic inflammation. On day 7, serum FITC-dextran fluorescence levels were significantly increased in DSS-treated mice compared with untreated mice (**Figure 5A**). Pre-treatment with DKT significantly suppressed serum FITC-dextran fluorescence levels to the levels seen in intact WT mice. In contrast, DKT treatment exhibited little inhibitory effects in SLPI^-/-^ mice (**Figure 5A**). Tight junction proteins (TJPs) are also important components of the intestinal epithelial barrier (27). Therefore, we examined the expression of occludin and ZO-1 proteins in the colon using immunohistochemistry, and found a decrease in their expression in the DSS-treated group compared to the DSS-untreated group (**Figure 5B**). Interestingly, pretreatment with DKT increased the expression of occludin and ZO-1 in DSS-treated WT mice, but not in the KO group (**Figure 5B**). These results indicate that DKT protects the intestinal epithelial barrier by maintaining TJPs in WT mice in an SLPI-dependent manner. Since neutrophil proteases are known to disrupt the intestinal epithelial barrier, we next evaluated the activity of neutrophil elastase (NE), a specific target of SLPI, in each mouse. NE activities in the colon were increased by DSS treatment, which was attenuated by DKT treatment in WT mice (**Figure 5C**). On the other hand, the suppressive effect was not seen in SLPI^-/-^ mice. These data suggest that DKT suppresses NE activity in neutrophils via the protease-inhibitory activity of SLPI.

### DKT alters the gut microbiota in an SLPI-dependent manner

**Figure 6:**
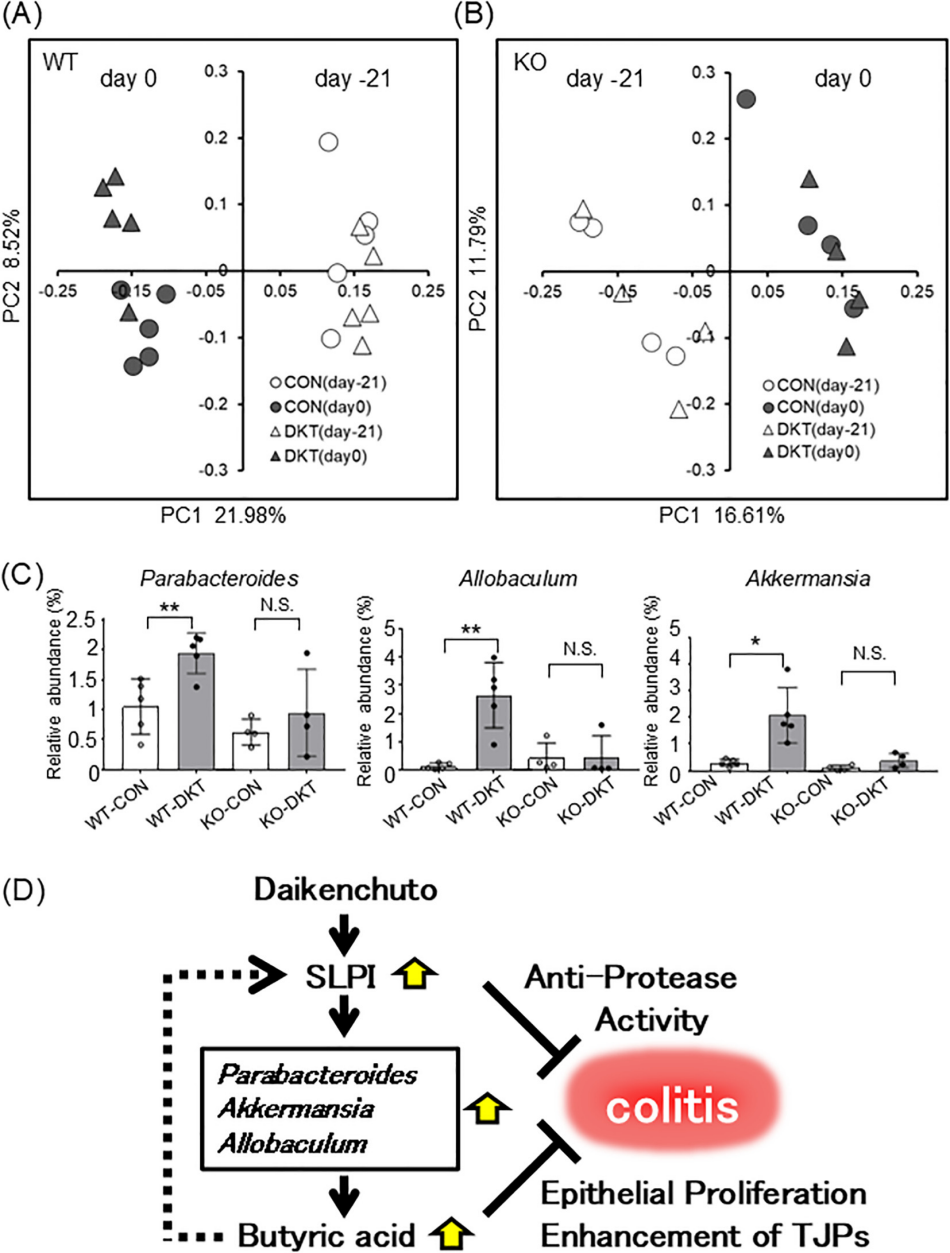
DKT alters the gut microbiota in an SLPI-dependent manner. **(A, B)** Visualization of principal coordinates analysis (PCoA) of unweighted UniFrac distances, to show differences in bacterial composition in WT **(A)** and SLPI^-/-^ mice **(B)**. Each point represents the fecal bacterial microbiota in a single sample. **(C)** Relative bacterial abundance is shown at the genus level. Data are presented as the mean ± SEM (WT: n = 5 in each group; SLPI^-/-^: n = 4 in each group) *: *P* < 0.05, **: *P* < 0.01, and NS, not significant. **(D)** Schematic model for the mechanism of DKT in suppressing colitis, whereby DKT induces SLPI, which in turn increases beneficial bacteria, such as *Parabacteroides*, *Allobaculum*, and *Akkermansia*, which produce butyric acid and further induce SLPI. SLPI inhibits neutrophil elastase activity and butyric acid enhances intestinal epithelial proliferation.

To investigate the impact of DKT and SLPI on the gut microbiota, we analyzed the composition of the gut microbiota by 16S rRNA metagenome sequencing of fecal samples. To assimilate the gut microbiota in a steady state, WT mice and SLPI^-/-^ mice were co-housed for several weeks prior to the experiment. 16S rRNA genes were sequenced and principal coordinate analysis (PCoA) plots were generated by QIIME 1.9.0 from MiSeq data. In WT mice, PCoA plots showed that each group formed a cluster of different intestinal flora after treatment with DKT in unweighted UniFrac distances (**Figure 6A**). On the other hand, PCoA plots showed that there was no obvious separation in SLPI^-/-^ mice with DKT treatment (**Figure 6B**). There was no significant difference in the profile of the gut microbiota in both WT and SLPI^-/-^ mice at the phylum level before administration of DKT (day -21) (**Supplementary Figure 2A**). However, after the DKT treatment (day 0), the abundance of beneficial bacteria, such as *Parabacteroides*, *Allobaculum*, and *Akkermansia*, was significantly increased at the genus level in WT mice, while there were no significant changes in these bacteria in SLPI^-/-^ mice (**Figure 6C**). These data indicate that DKT might alter the colonic bacterial community in an SLPI-dependent manner.

We next analyzed the composition of the gut microbiota in mice with colitis (day 7). Alpha diversity analysis revealed that DSS treatment reduced microbial diversity in fecal samples from both WT and SLPI^-/-^ mice (**Supplementary Figure 2B**). DKT suppressed the reduction of alpha diversity by DSS treatment in WT mice, but not in SLPI^-/-^ mice (**Supplementary Figure 2B**). In beta diversity analysis, PCoA plots were not clearly separated after DSS treatment regardless of dietary DKT intake (**Supplementary Figure 3A**). Genus-level profiling showed that DSS treatment increased the relative abundance of *Turicibacter*, also known as colitogenic bacteria, in both WT and SLPI^-/-^ mice, and no significant effect of DKT was observed (**Supplementary Figure 3B**). Moreover, there were no significant changes in the gut microbiota at the genus level between DKT treated and untreated groups of DSS-treated mice (day 7), probably due to limited activity of DKT under DSS-induced inflammation in the colon (**Supplementary Figure 4**).

## Discussion

In the present study, we showed that SLPI, directly induced by DKT in the colon, as well as indirectly by modulating the gut microbiota and increasing butyrate production, alleviates DSS-induced colitis by protecting the intestinal epithelial barrier. Our findings delineate the molecular mechanisms by which DKT ameliorates IBD.

IBD, such as CD and UC, is characterized by chronic relapsing inflammation of the intestine. Although the precise mechanism of IBD remains unclear, dysfunction of the intestinal epithelial barrier is crucial for the development and perpetuation of IBD (26). Once the intestinal barrier function is disrupted by genetic or environmental factors, bacterial antigens in the intestinal tract come into contact with immune cells, leading to unwanted immune reactions and an excessive recruitment of neutrophils to sites of inflammation (28, 29). Eventually, the intestinal epithelium is further damaged by the neutrophil proteases released from neutrophils (30). Thus, neutrophil proteases must be strictly controlled to ensure the integrity of the intestinal epithelial barrier.

DKT, a traditional Japanese herbal medicine, has been shown to increase intestinal blood flow and improve intestinal propulsive motility, as well as to have anti-inflammatory effects in experimental models. For example, Matsunaga et al. revealed that DKT attenuated DSS-induced colitis by enhancing the anti-inflammatory cytokine IL-10 (13). Furthermore, Li et al. have reported that DKT reduces methotrexate-induced small intestinal inflammation by stimulating cell proliferation and protecting the intestinal barrier (12). In the present study, we found that SLPI was induced by DKT in a colon carcinoma cell line *in vitro*, and in the colon of mice *in vivo* (**Figures 2**, **3**; **Supplementary Figure 1B**). SLPI is an endogenous serine protease inhibitor expressed at mucosal surfaces, mainly by epithelial cells (31). We previously showed that the protease inhibitory activity of SLPI prevents the intestinal epithelial barrier dysfunction caused by excessive NE activity in DSS-induced colitis (21). Thus, SLPI maintains homeostasis of the intestinal barrier by preventing tissue destruction and regulating the threshold of inflammatory immune responses in the intestine. In this study, we revealed that administration of DKT attenuates DSS-induced colitis in WT mice, but not in SLPI^-/-^ mice (**Figure 3**). In particular, increased permeability of the epithelium caused by DSS treatment was suppressed by DKT in an SLPI-dependent manner (**Figure 5A**). This is probably partly due to the fact that DKT administration maintained the expression of the adhesion molecules, occludin and ZO-1, under DSS-induced inflammation in the colon of WT mice, but not in SLPI^-/-^ mice (**Figure 5B**). These results indicate that DKT attenuates DSS-induced colitis by protecting the intestinal epithelial barrier in an SLPI-dependent manner. Infiltration of neutrophils into the mucosal tissue is a hallmark of colitis activity. Subsequently, neutrophil proteases, such as NE, derived from neutrophils damage the gut epithelium, resulting in increased permeability (30). Given the protease inhibitory activity of SLPI, it is likely that administration of DKT might inhibit the activity of NE that is enhanced by DSS-induced colitis. As expected, the activity of MPO, an indicator of neutrophil activation, and NE activity were decreased by DKT treatment in WT mice, but not in SLPI^-/-^ mice (**Figures 4D**, **5C**). These results suggest that DKT exerts protective effects against colitis due, at least in part, to induction of anti-protease protein SLPI in the colon.

Interactions between various natural compounds or diets and the gut microbiota are known to alter intestinal permeability and the severity of colitis in mice (32, 33). DKT, a combination of four natural herbs, has been reported to alter the microbiota composition in mice in a dose-dependent manner (16). Long-term dietary DKT increases the abundance of a number of bacteria that produce SCFAs, including butyrate, propionate, and acetate (15). On the other hand, SLPI is known to have broad spectrum antimicrobial activity against enteric pathogens, including Gram-positive and -negative bacteria and fungi (34). We, therefore, explored the possible involvement of DKT and SLPI in the regulation of the gut microbiota composition, and its effect on DSS-induced colitis. In the principal coordinates analysis, the composition of the gut microbiota was clearly separated between pre- and post-DKT treatment of mice in the presence of SLPI (**Figure 6A**). In particular, administration of DKT increased the relative abundance of the beneficial bacteria, *Parabacteroides*, *Allobaculum*, and *Akkermansia* at the genus level (**Figure 6C**). These changes were only seen in WT mice, suggesting that DKT might alter the gut microbiota composition in an SLPI-dependent manner. SLPI has been known to have not only an inhibitory property against serine proteases but also an antimicrobial property against Gram-negative bacteria *in vitro* (34). It has been reported that deficiency of certain antimicrobial peptides can alter the occupancy of specific commensal bacteria and change the composition of the intestinal microbiota (35). Therefore, the lack of SLPI may influence to certain commensal bacteria in the gut. In addition, it has been reported that DKT increases IL-22 following ILC3 induction (17) and that IL-22 increases certain commensal intestinal bacteria (*Akkermansia*) by induction of antimicrobial peptides (36). Thus, SLPI induced by DKT, may control the growth of specific commensal bacteria such as *Akkermansia* through its antimicrobial activity. However, the precise mechanism has not yet been elucidated in this study, and is a subject for future research.

*Parabacteroides* spp., core members of the human gut microbiota, have a close relationship with host health due to its physiological characteristics on carbohydrate metabolism and secreting SCFAs (37). A previous animal study has reported that oral administration of *Parabacteroides distasonis* and its cellular components attenuate experimental colitis through the reduction of pro-inflammatory cytokines and increase in the number of regulatory T cells in the colon (38). *Allobaculum* spp., known as a butyric acid producer, is a newly identified IBD-associated bacteria that is thought to be closely associated with the host epithelial barrier (39, 40). *Akkermansia* spp. reportedly ameliorate the symptoms of DSS-induced colitis through the upregulation of NLPR3 (41). Moreover, *Akkermansia* species enhance the function of the intestinal epithelial barrier by producing mucin and TJPs in intestinal epithelial cells (42, 43). Since the initial gut microbiota has a profound influence on DSS-induced colitis (44), greater abundance of beneficial bacteria, such as *Parabacteroides*, *Allobaculum*, and *Akkermansia*, by treatment with DKT might reduce the susceptibility to DSS-induced colitis. Moreover, these bacteria are known to produce butyric acid (39, 45, 46), which is consistent with our observation that DKT treatment increased both, the relative abundance of these bacteria (**Figure 6C**) and the amount of butyric acid in the colon (**Figure 2D**). Interestingly, butyrate enhanced the expression of SLPI in a colon carcinoma cell line *in vitro* (**Figure 2E**), suggesting that DKT increases butyrate-producing bacteria via induction of SLPI expression, which forms a positive feedback loop whereby butyrate induces further SLPI expression (**Figure 6D**). In addition, the expression of SLPI was not increased by DKT treatment in antibiotics-treated mice (**Supplementary Figure 3C**), suggesting that gut microbiota is required for the proper induction of SLPI. In other word, DKT directly induces SLPI from intestinal epithelial cells *in vitro*, but requires the presence of intestinal bacteria for the proper induction of SLPI *in vivo*. SCFAs, such as butyric acid, are known to enhance epithelial integrity by enhancing intestinal cell proliferation (47) and modulating TJP expression (48, 49), suggesting that increased butyric acid contributes to ameliorating DSS-induced colitis.

We found that abundance of the genus *Turicibacter*, known to correlate positively with the development of IBD (50), was increased by DSS treatment in the presence of SLPI, while DKT treatment failed to inhibit its growth (**Supplementary Figure 3B**). These results indicate that the effect of DKT on the gut microbiota composition is limited to bacterial species.

Although the present findings provide insight into the mechanism of the colon protective effect of DKT via induction of SLPI, the precise signaling pathways by which DKT induces SLPI remain unclear. In general, herbal medicines consist of multiple plant extracts containing several constituents, which are often believed to work together synergistically (51). The pharmacological effects of DKT were not analyzed at the component level in this study. Therefore, determining the biological components of DKT that induce SLPI and modulate the gut microbiota would be of interest for future research.

In conclusion, DKT exerts a protective effect against DSS-induced colitis, in which SLPI promotes prebiotic effects by reshaping the gut microbiota and increasing butyrate-producing bacteria, while enhancing the integrity of the intestinal epithelial barrier through protease inhibitory activity. Therefore, our findings shed new light on the mechanism of the therapeutic effect of DKT on IBD.

## Materials and methods

### Drugs and chemicals

Daikenchuto (DKT, TJ-100) was obtained from Tsumura & Co. (Ibaraki, Japan). DKT consists of an herbal medicinal extract powder (DKT-E) and maltose (ratio 1.25 g to 10 g, respectively). DKT-E was prepared by spray-drying a hot-water extract mixture containing the following three crude drugs/herbs in the proportions mentioned in parentheses: *Zanthoxyli fructus* (Japanese pepper) (2.0), *Zingiberis rhizoma* (Ginger) (5.0), and *Panax ginseng* (Ginseng radix) (3.0). DKT was included in PicoLab rodent diet 20 (Land O’ Lakes, Inc., Arden Hills, MN, USA), at 36 g DKT/kg of diet (3.6% wt/wt) (Oriental Yeast Co., Ltd., Tokyo, Japan). The dosage of DKT was based on a previous animal experiment (16) and the human equivalent dosage was adjusted to the mouse dosage based on the animal’s surface area (52). Ampicillin and vancomycin were purchased from Wako (Osaka, Japan).

### Cell culture and CRISPR/Cas9

CMT93, a mouse colon carcinoma cell line, was purchased from ECACC (Salisbury, UK). CMT93 cells were cultured in Dulbecco’s modified Eagle medium (DMEM) containing 10% FCS, 2 mM L-Alanyl-L-glutamine (Nacalai tesque), 100 U/mL penicillin, and 100 μg/mL streptomycin (Nacalai tesque, Kyoto, Japan). We established TNF receptor-associated factor 6 (TRAF6) knockout (KO) CMT93 cells using the CRISPR-Cas9 genome editing system. Briefly, a single guide RNA targeting TRAF6 (5’-GGAGGACAAGGTTGCCGAAA-3’) was cloned into a Cas9-expressing plasmid (pSpCas9(BB)-2A-Puro (PX459); addgene plasmid #48139), and the plasmid was transfected into CMT93 cells, and then subsequently cloned after a 2-day treatment with 2 μg/mL puromycine. DKT-E was suspended in distilled water, sterilized by boiling at 95°C, passed through a 0.45 μm filter, and then added to CMT93 cells at final concentrations of 330 or 1000 μg/mL. In addition, CMT93 cells were treated with 1 mM sodium butyrate (Wako Pure Chemical Industries, Ltd., Kyoto, Japan) or stimulated with 3 μg/mL of LPS (Sigma-Aldrich, St. Louis, MO, USA) for 24 hours. The cells were then harvested and total RNA was extracted using TRI Reagent^®^ (Sigma-Aldrich, St. Louis, MO, USA) for performing quantitative RT-PCR analysis.

### Animals and ethics statement

Seven-week-old female C57BL/6 mice were purchased from Japan SLC (Hamamatsu, Japan). SLPI^-/-^ mice, as described previously (20), were backcrossed with C57BL/6 inbred mice more than 10 times. Mice were maintained in a specific pathogen-free facility under conditions of constant temperature (24 ± 1°C), humidity (50 - 60%), and a 12-hour light-dark cycle with free access to food and water. All experiments using these mice were approved by and performed according to the guidelines of the Oita University Animal Ethics Committee (approval number: 180901A). This study adheres to standards articulated in the ARRIVE guidelines.

### Study design and colitis induction

A 28-day protocol was designed and mice were fed a normal diet or a 3.6% DKT diet during this protocol period (day -21 to 7). To induce colitis, mice were administered 2% Dextran Sulfate Sodium Salt (DSS) - Colitis Grade (36,000-50,000 MW) (MP Biomedicals, Irvine, CA, USA) in drinking water for 5 days (days 1-5), followed by normal water for 2 days (days 6-7). Their body weight and disease activity index (DAI) were monitored daily. DAI scores were determined according to a previous report (53) as follows: body weight loss (0: no loss; 1: 1-5%; 2: 5-10%; 3: 10-20%; 4: >20% loss), stool consistency (0: normal; 2: loose stools; 3: mud stools; 4: diarrhea), and bleeding per rectum (0: no blood; 2: visual pellet bleeding; 3: blood around anus; 4: gross bleeding).

### Histological analysis

Mice were sacrificed by cervical dislocation on day 28 and their large intestine was removed. After measuring the length of the colon, the distal parts of the colon were stained with hematoxylin and eosin (H&E). The severity of DSS-induced colitis was evaluated using distal colon sections by a modified histological scoring system (54) as follows: epithelial cell damage (0: no damage; 1: focal loss of goblet cells; 2: diffuse loss of goblet cells; 3: focal loss of crypts; 4: diffuse loss of crypts), cell infiltration (0: no increase; 1: around the base of the crypts; 2: along the muscularis mucosal layer; 3: mucosal layer; 4: mucosal and submucosal layer), ulcer (0: no ulcer; 1: focal erosion; 2: diffuse shallow ulcer on the epithelial surface or focal ulcer in the mucosal layer; 3: diffuse ulcer involving the entire mucosal layer). Immunohistochemistry was performed using 2 μm sections of paraffin-embedded colon tissue. We used primary antibodies against occludin (Abcam, Cambridge, UK), ZO-1 (Gene Tex, Irvine, CA, USA), and SLPI (R&D Biosystems, Minneapolis, MN, USA). DAKO EnVision™+ (Rabbit) (Agilent Technologies) was used as a secondary antibody. Sections were subsequently counterstained with hematoxylin. All histological evaluations were performed in a blind fashion.

### Gas chromatography-mass spectrometry analysis

Metabolites in the cecal contents were analyzed by GC/MS-TQ8040 (Shimazu, Kyoto, Japan) with a BPX-5 column (30 m × 0.25 mm i.d.; film thickness 1.00 μm, Trajan Scientific and Medical, Vic., Australia) for SCFAs, as described previously (55). Mass spectrum peaks were detected using the GC/MS solution software (Shimazu), and the retention time correction of peaks was performed based on the retention time of a standard alkane series mixture (C9 to C33). Metabolites were identified by the Smart Metabolites Database (Shimazu), which contains multiple reaction monitoring transitions for 12 metabolites commonly found in biological samples for BPX-5. Raw data have been deposited at figshare with DOI: 10.6084/m9.figshare.26212430.

### DNA extraction and 16S rRNA gene metagenome sequencing analysis

The bacterial genomic DNA was isolated using the standard protocol with some modifications (56). DNA from mouse feces was extracted using a phenol/chloroform/isoamyl alcohol method. Preparation of the 16S rRNA gene metagenome library for MiSeq (Illumina, Inc., San Diego, CA, USA) was performed according to the manufacturer’s protocol. Briefly, 10 ng of the DNA template was amplified using an Advantage-HF 2 PCR kit (Takara Bio Inc., Shiga, Japan) with universal primers for the 16S rRNA v3-v4 region (forward primer: 5’ TCGTCGGCAGCGTCAGATGTGTATAAGAGACAGCCTACGGGNGGCWGCAG 3’, reverse primer: 5’ GTCTCGTGGGCTCGGAGATGTGTATAAGAGACAGGACTACHVGGGTATCTAATCC 3’). Subsequently, index sequences for each sample were added to both ends of the purified PCR fragments. The concentrations of each amplicon were measured by the Quant-iT PicoGreen dsDNA Assay Kit (Thermo Fisher Scientific, Inc.) and mixed equally. The library was applied to MiSeq Reagent Kit v3 (Illumina, Inc.) and the sequence was determined using the manufacturer’s standard protocol. Sequence data were processed as follows using the 16S rRNA sequence analysis pipeline, QIIME 1.9.0 (57). Initially, both sequence reads were joined and sequences with a Phred quality score below 20 were removed. Chimera elimination by Usearch was performed to remove contaminated sequences. Open reference OTU picking was performed against Greengenes 13_8 97% OTU representative sequences. A summary of taxonomy in each sample was obtained using the script ‘summarize_taxonomy_through_plots.py’ in QIIME 1.9.0. Raw data have been deposited at figshare with DOI: 10.6084/m9.figshare.26241974.

### Measurement of intestinal permeability

Mice were fasted for 4 hours and administered FITC-dextran (4 kDa MW, 0.6 mg/g body weight) (Sigma-Aldrich) by oral gavage. Four hours later, blood samples were collected and the fluorescence intensity of serum FITC at 485/528 nm wavelength was measured using a microplate reader (Infinite 200 PRO, TECAN, Männedorf, Switzerland).

### Myeloperoxidase assay and neutrophil elastase activity assay

MPO activity was determined in colon tissues. Briefly, the colon tissues were homogenized in 500 μL of potassium phosphate buffer (pH 6.0) containing 0.5% hexadecyltrimethylammonium bromide (HTAB) (Sigma-Aldrich). Then, the samples were centrifuged at 14,600 rpm for 15 min at 4°C. Thereafter, 7 μL of the supernatant was mixed with 200 μL of O-dianisidine/Buffer solution, and absorbance readings at 450 nm were assessed every minute for 30 minutes. Colonic NE activity was measured using a Neutrophil Elastase Activity Assay Kit, Fluorometric (Abcam), according to the manufacturer’s instructions. The colon tissues were homogenized in 500 μL of HTAB buffer and then incubated with substrate in reaction buffer. Fluorescence was determined using a microplate reader at 380/500 nm wavelength every 3 minutes for 30 minutes.

### Western blot analysis

Total proteins in the colons were subjected to SDS-PAGE. The separated protein on a PVDF membrane (Millipore, Darmstadt, Germany) was incubated with primary antibody against SLPI (1: 1000; R&D Biosystems) overnight at 4°C, followed by secondary antibody conjugated with horseradish peroxidase, and visualized with the ECL Western blotting analysis system (GE Healthcare, Piscataway, NJ, USA). Levels of SLPI were measured by quantification of the band intensities with ImageQuant TL software (Roche Diagnosis, Rotkreuz, Switzerland).

### Quantitative real-time reverse transcription polymerase chain reaction

Total RNA from the colon was extracted using TRI Reagent^®^, purified using a PureLink RNA Mini Kit (Thermo Fisher Scientific Inc.) and then reverse-transcribed using a Verso cDNA Synthesis Kit (Thermo Fisher Scientific Inc.). Quantitative RT-PCR was performed using a real-time PCR machine (LightCycler 96, Roche Diagnostics) with a KAPA SYBR FAST qPCR Kit (Kapa Biosystems, Wilmington, MA, USA). The relative mRNA levels were normalized to β-actin, and data were analyzed by LightCycler software (Roche Diagnostics). The sequences of primers used in this study are shown in **Table 1**.

**Table 1:** Primer sequences used for polymerase chain reaction in this study.

Target	Forward 5’-3’	Reverse 5’-3’
m β-actin	CTTCCTCCCTGGAGAAGAGCTATGAGC	GCCTAGAAGCACTTGCGGTGCACG
m SLPI	GGCCTTTTACCTTTCACGGTG	TACGGCATTGTGGCTTCTCAA
m BD3	CTTTGCATTTCTCCTGGTGC	GCCTCCTTTCCTCAAACT
m Lactoferrin	TCAAGAAATCCTCCACCCGC	ACACGAGCTACACAGGTTGG
m Reg3γ	TTCCTGTCCTCCATGATCAAAA	CATCCACCTCCGTTGGGTTCA
m Spink4	AGGGAACTGATGGTGTCAGC	AGATCAGGTTGGGTGTCTGG
m Serpina3N	ATTTGTCCCAATGTCTGCGAA	TGGCTATCTTGGCTATAAAGGGG
m IL-1β	GAGTGTGGATCCCAAGCAAT	TACCAGTTGGGGAACTCTGC
m IL-23p19	CACAGAGCCAGCCAGATCTGAGAAGC	CCATGGGAACCTGGGCATCCTTAAGC
m IL-6	CCGGAGAGGAGACTTCACAG	CAGAATTGCCATTGCACAAC
m IFN-γ	ATGAACGCTACACACTGCATC	CCATCCTTTTGCCAGTTCCTC
m IL-17A	GGCCCTCAGACTACCTCAACC	TGAGCTTCCCAGATCACAGAG

### Statistical analysis

All data are presented as the mean ± SEM. Differences between two groups were analyzed by Student’s *t* test. Multiple comparisons were analyzed by one-way ANOVA followed by Tukey’s multiple comparisons test. Alpha and beta diversity analyses in fecal samples were calculated using QIIME 1.9.0. Univariate analysis between two groups was performed with the Mann-Whitney *U* test using GraphPad Prism7 software (GraphPad Software, San Diego, CA, USA). The graphs were visualized by Excel Software and GraphPad Prism 7 software. *P* values less than 0.05 were considered statistically significant in all experiments. In the figures, *: *P* < 0.05, **: *P* < 0.01, ***: *P* < 0.001, and ****: *P* < 0.0001.

## Data Availability

The raw data supporting the conclusions of this article will be made available by the authors, without undue reservation.

## Supplementary material

Supplementary Figure 1
**(A)** Quantitative RT-PCR analysis of the mRNA expression of SLPI in the colon of mice after oral butyrate supplementation. Graphs show the mean ± SEM (n = 5). **(B)** Immunohistochemistry of SLPI in the colon tissues from WT mice treated with normal or DKT diet for 28 days (upper panel: WT-CON, middle panel: WT-DKT). A higher magnification image of the rectangle in the middle panel is shown in the lowest panel. The scale bar represents 50 μm (upper and middle panels) and 10 μm (lowest panel).

Supplementary Figure 2
**(A)** Fecal samples from mice before the DKT treatment (on day -21) were subjected to 16S rRNA metagenome sequencing to examine the composition of the gut microbiota. The relative abundance of bacteria at a phylum level is shown. Each bar shows relative bacterial abundance in individual mice (WT mice: n = 10, SLPI-/- mice: n = 8). **(B)** Alpha diversity of the gut microbiota is shown. Data are presented as the mean ± SEM (WT: n = 5, SLPI-/-: n = 4 in each group). *: P < 0.05 and NS, not significant.

Supplementary Figure 3
**(A)** Visualization of principal coordinates analysis (PCoA) of unweighted UniFrac distances showing differences in bacterial composition. Each point represents the fecal bacterial microbiota in a single sample. **(B)** The relative abundance of Turicibacter is shown. Data are presented as the mean ± SEM (WT: n = 5 in each group; SLPI-/-: n = 4 in each group). **(C)** 1 g/L ampicillin and 0.5 g/L vancomycin were administered in drinking water for seven days to eliminate bacteria in the intestine. Quantitative RT-PCR analysis of the mRNA expression of SLPI in the colon of antibiotics-treated WT mice after the treatment of normal diet (antibiotic group) or DKT diet (antibiotic + DKT group). Graphs show the mean ± SEM (n = 4). NS, not significant.

Supplementary Figure 4 Fecal samples from mice fed a normal diet or DKT diet for 21 days following DSS treatment for 7 days (on day 7) were subjected to 16S rRNA metagenome sequencing to examine the composition of the gut microbiota. The relative abundance of bacterial genera is shown. Each bar shows relative bacterial abundance in individual mice (WT: n = 5 in each group; SLPI-/-: n = 4 in each group).
