# Supplementary figures and images for: Daikenchuto, a Japanese herbal medicine, ameliorates experimental colitis in a murine model by inducing secretory leukocyte protease inhibitor and modulating the gut microbiota
Source: Front Immunol. 2024 Oct 25;15:1457562. doi: 10.3389/fimmu.2024.1457562 (PMC11543465; doi:10.3389/fimmu.2024.1457562)

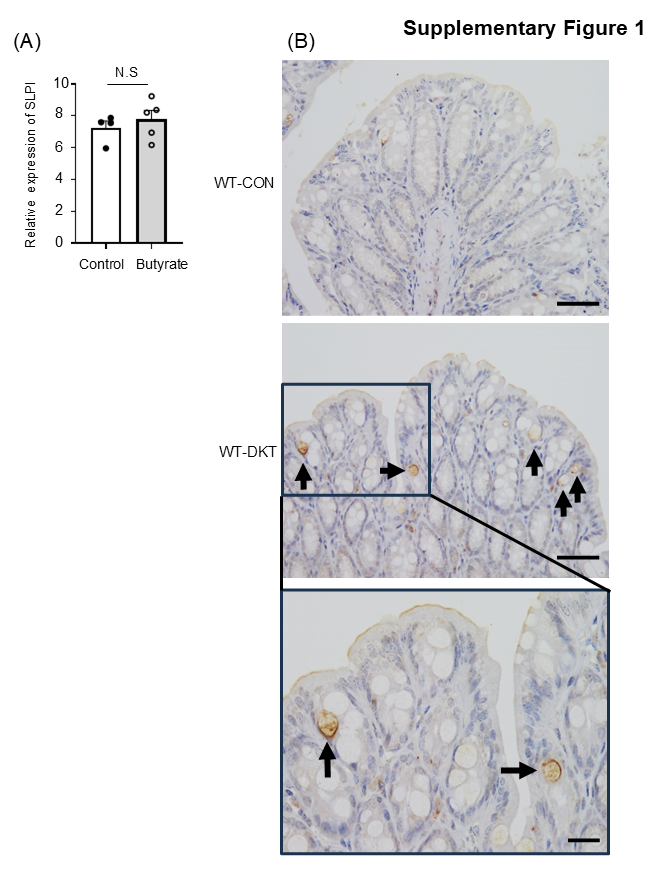

Supplement: Supplementary Figure 1 — (A) Quantitative RT-PCR analysis of the mRNA expression of SLPI in the colon of mice after oral butyrate supplementation. Graphs show the mean ± SEM (n = 5). (B) Immunohistochemistry of SLPI in the colon tissues from WT mice treated with normal or DKT diet for 28 days (upper panel: WT-CON, middle panel: WT-DKT). A higher magnification image of the rectangle in the middle panel is shown in the lowest panel. The scale bar represents 50 μm (upper and middle panels) and 10 μm (lowest panel). [file Image1.tif]

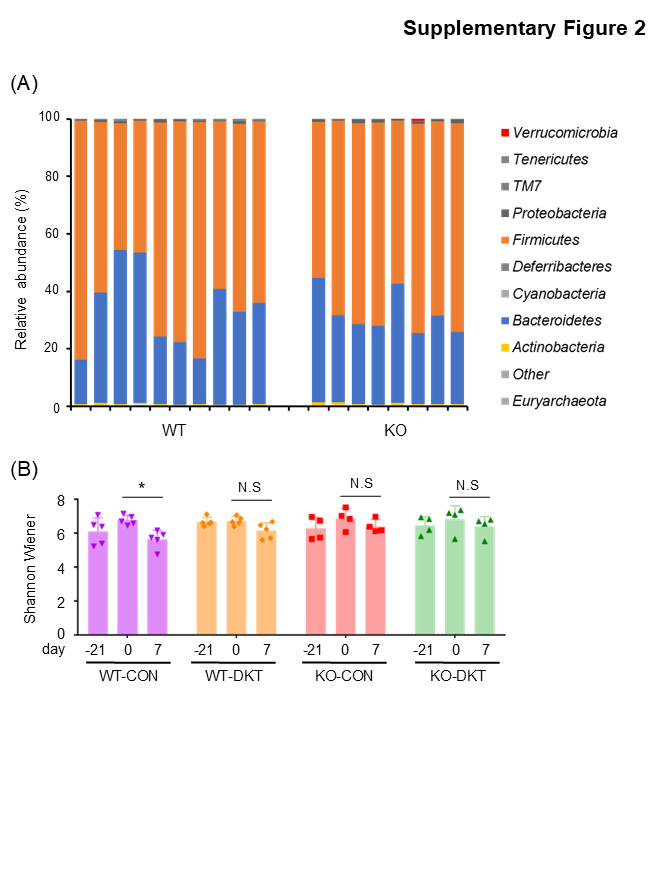

Supplement: Supplementary Figure 2 — (A) Fecal samples from mice before the DKT treatment (on day -21) were subjected to 16S rRNA metagenome sequencing to examine the composition of the gut microbiota. The relative abundance of bacteria at a phylum level is shown. Each bar shows relative bacterial abundance in individual mice (WT mice: n = 10, SLPI-/- mice: n = 8). (B) Alpha diversity of the gut microbiota is shown. Data are presented as the mean ± SEM (WT: n = 5, SLPI-/-: n = 4 in each group). *: P < 0.05 and NS, not significant. [file Image2.tif]

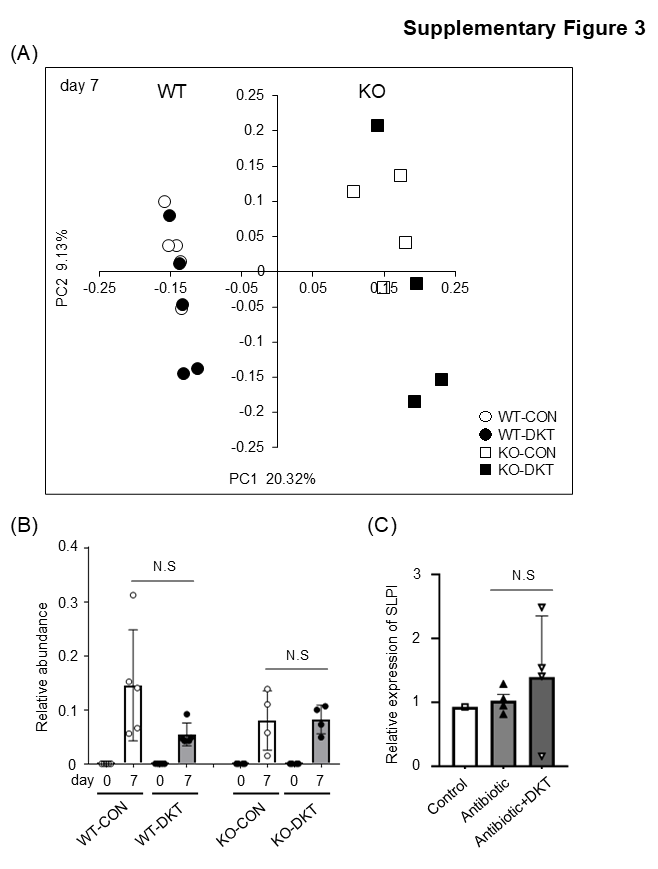

Supplement: Supplementary Figure 3 — (A) Visualization of principal coordinates analysis (PCoA) of unweighted UniFrac distances showing differences in bacterial composition. Each point represents the fecal bacterial microbiota in a single sample. (B) The relative abundance of Turicibacter is shown. Data are presented as the mean ± SEM (WT: n = 5 in each group; SLPI-/-: n = 4 in each group). (C) 1 g/L ampicillin and 0.5 g/L vancomycin were administered in drinking water for seven days to eliminate bacteria in the intestine. Quantitative RT-PCR analysis of the mRNA expression of SLPI in the colon of antibiotics-treated WT mice after the treatment of normal diet (antibiotic group) or DKT diet (antibiotic + DKT group). Graphs show the mean ± SEM (n = 4). NS, not significant. [file Image3.tif]

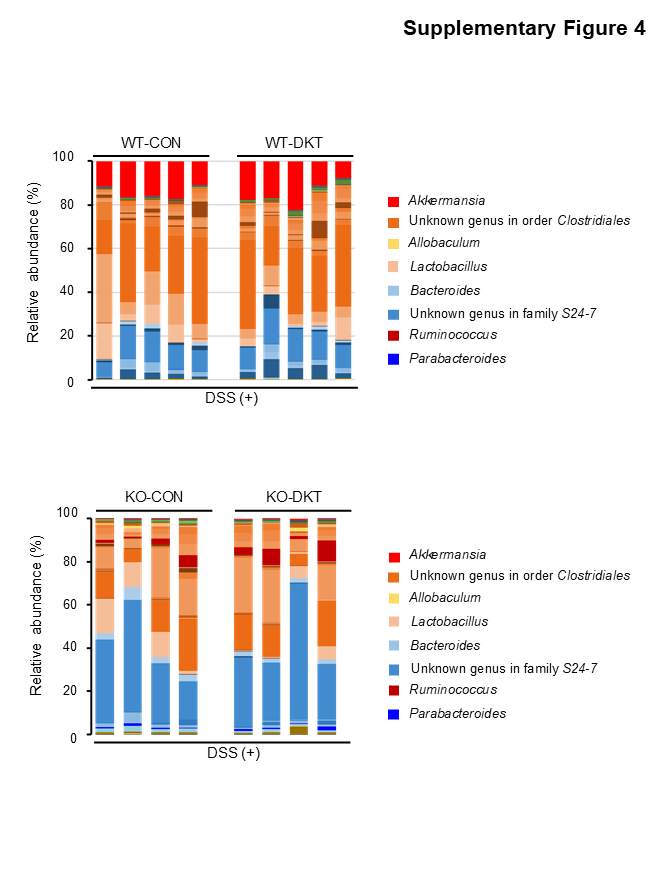

Supplement: Supplementary Figure 4 — Fecal samples from mice fed a normal diet or DKT diet for 21 days following DSS treatment for 7 days (on day 7) were subjected to 16S rRNA metagenome sequencing to examine the composition of the gut microbiota. The relative abundance of bacterial genera is shown. Each bar shows relative bacterial abundance in individual mice (WT: n = 5 in each group; SLPI-/-: n = 4 in each group). [file Image4.tif]
